# Navigating ‘Home Schooling’ during COVID-19: Australian public response on Twitter

**DOI:** 10.1177/1329878X20956409

**Published:** 2021-02

**Authors:** Lee-Ann Ewing, Huy Quan Vu

**Affiliations:** Monash University, Australia; Deakin University, Australia

**Keywords:** COVID-19, pandemic, remote learning, social media analytics, Twitter

## Abstract

COVID-19 has wreaked havoc worldwide. Schools have escaped neither the pandemic nor its consequences. Indeed, by April 2020, schools had been suspended in 189 countries, affecting 89% of learners globally. While the Australian government has implemented variously effective health and economic policies in response to COVID-19, their inability to agree with states on education policy during the pandemic caused considerable confusion and anxiety. Accordingly, this study analyses 3 weeks of Tweets during April, leading up to the beginning of term 2, during the height of Government policy incongruity. Findings confirm a wide and rapidly changing range of public responses on Twitter. Nine themes were identified in the quantitative analysis, and six of these (positive, negative, humorous, appreciation for teachers, comments aimed at Government/politicians and definitions) are expanded upon qualitatively. Over the course of 3 weeks, the public began to lose its sense of humour and negative tweets almost doubled.

## Introduction

On 31 December 2019, the first reporting of unusual health activity came out of Wuhan, in Hubei Province, China. Eight days later, the activity was identified as a ‘novel coronavirus’ (virus strain SARS-CoV-2), named 2019-nCoV or COVID-19 by the World Health Organization (WHO, CNN Editorial Research, 2020). Over the next several weeks, cases began to grow in Asia, particularly China. As the world watched government actions and news updates out of China, Thailand, Japan and South Korea, the virus began to make its way across the globe. Given its spread, mortality rate and global impact, on 11 March, the WHO declared the outbreak a pandemic.

On 1 March 2020, Australia reported the first death from COVID-19: a 78-year-old Perth man, who was one of the passengers from the *Diamond Princess* cruise ship (ABC, 2020). Consequently, Australian borders were closed to all non-residents on 20 March (Burke, 2020). Social distancing rules were imposed on 21 March and the state governments started to close ‘non-essential’ services thereafter. As of the end of 6 April, 753 cases had been reported in Australia. The Australian Federal Government’s pandemic suppression strategy is potentially the envy of many nations (albeit the same might not be said of the Victorian state government’s response). Indeed, by April, Australia has conducted more tests and registered less cases and less deaths per capita than most other countries. Similarly, the Australian government’s economic stimulus programme is the third largest in the world (behind Japan and the United States), at 10% of GDP by April. For the most part, these policies received bipartisan support at both federal *and* state levels. However, the same cannot be said of the Federal Government’s attempts at formulating educational policies with regard to school attendance, social distancing and remote learning.

The COVID-19 pandemic of 2020 has necessitated unprecedented political and policy agility. The issue of whether to keep schools open or not has continued to vex politicians and perplex the public. Confusion has been exacerbated by divisions and differences of opinion within and between the federal government and state premiers. To illustrate the confusion, the Federal Education Minister was still undecided on whether or not his own children would attend school (in Victoria), even on the eve of term 2 commencing after the Easter vacation: ‘My children at this stage, their school doesn’t start till tomorrow. I’ll be continuing to have discussions with them about that. We’ll wait and see what happens’. Mr. Tehan denied that his own indecision mirrored parents’ confusion. Prime Minister Scott Morrison has been consistent in his personal and professional preference, signalling his desire to send his own daughters back to school during the COVID-19 pandemic, as long as ‘proper teaching resumes in Australia’s classrooms’. In an interview on 15 April, the prime minister explained he would have his children back in classrooms ‘in a heartbeat’ – if schools were back to ordinary lessons: ‘I kept my kids in school up until the last week because they weren’t getting taught at school in that last week’, he said on 6PR. ‘I mean, they were sitting in a room looking at a screen, that’s not teaching, that’s childminding. And schools aren’t for childminding. Schools are for teaching and they’re for learning’.

All states and territories, except the Northern Territory where attendance is compulsory, have made school optional. Some, including Victoria, have told parents not to send children to class during the coronavirus pandemic. See Table 1 below – as at the end of April 2020.

**Table 1.** Australian state and territory policies at the beginning of term 2.

State (political party)	Policy* (as on 26 April)	COVID cases per 100,000 (27 April)**
ACT (Liberal)	School will return in the ACT on Tuesday (28/4), and **will initially be entirely delivered via remote learning.**	25
NSW (Liberal)	**Staggered return to face-to-face classroom teaching through 5 phases.** Phase 0 sees all students encouraged to utilise remote learning from home wherever possible, though ‘no student will be turned away’ from supervised school learning. **Phase 1 begins May 11**, requires students to return to the classroom for day/week. Phase 2 = 2 days/week. Phase 3 = 5 days/week with social distancing. Phase 4 would have school back running as normal.	**37**
WA (Labor)	Schools **open for any student to receive face-to-face teaching.** WA Government claim term two plan is ‘cautious, careful and considered to protect teachers, parents and children’. Choice to send children to school lies with families, and distance education packages and resources or online remote learning will be provided to any student who is kept home. **Year 11 and 12 students** ‘**strongly encouraged**’ **to attend school** for face-to-face classes. Some Catholic, independent and Anglican schools have gone against this advice, adopting remote learning for students up to Year 10.	21
NT (Labor)	All students in the **NT are expected to physically attend school, unless they are unwell.** Parents can choose not to send their children to school, but are then ‘responsible for the student’s learning, safety and wellbeing at home or elsewhere’.	11
Queensland (Labor)	Queensland schools also reopened last week, with **remote learning currently in place for at least the first half of term two, until May 22.** Queensland Government’s policy is ‘all students who are able to be supervised at home and learn from home are to stay home, except for vulnerable students and children of essential workers’	20
South Australia (Liberal)	Late notification came through that **schools *would* be open and students should attend.** Remote learning is available for students who are kept at home, but Premier Steven Marshall said ‘**we would like to see your attendance in term two and beyond**’.	25
Tasmania (Liberal)	**Students have been encouraged to learn from home wherever possible.** While schools will be open through most of the state for students who for whatever reason are unable learn from home.	**40**
Victoria (Labor)	Victorian Government has been the firmest of the seven in its stance that **students must learn from home if possible.** Schools are open for those who are unable to utilise remote learning but, despite some pressure from the opposition, the **Government intends for all of term two to be delivered remotely.**	20

* https://www.abc.net.au/news/2020-04-27/how-schools-in-states-and-territories-term-two-coronavirus/12186022

** https://www.statista.com/statistics/1103944/australia-coronavirus-cases-per-100-000-population-by-state/

In an attempt to expedite its wish for all pupils to physically return to the classroom, the Federal Government set aside three billion dollars in incentives in late April to encourage independent schools to reopen classrooms (Carey and Fowler, 2020). However, this incentive was been ignored by most Catholic and independent schools – albeit not without causing them considerable stress. Indeed, the Federal-State tension is most pronounced in Victoria, Australia’s second most populous state (6.49 million). This tension reached boiling point on 3 May, when the Federal Education Minister attacked the Victorian premier (Dan Andrews) for ‘taking a sledgehammer to the state’s education sector’, before accusing Mr. Andrews of ‘a failure of leadership’ (Worthington, 2020). Within a matter of hours, the Federal Education Minister withdrew his comments in a press release.

Elected officials attempted to influence how citizens think about problems and solutions through speeches, news conferences, press releases, web and social media posts. The public, typically, use social media to voice their reactions to government policies. In an attempt to better understand public sentiment in response to this situation, this study reports on a content analysis of Australian-based Tweets (#homeschooling) between April and 3 May (up to and including the start of term 2).

## Methodology

The data were collected from Twitter, one of the most popular social networking platforms worldwide. Twitter allows users to post and interact with messages know as tweets. Users often access the Twitter platform through its web interface, or its mobile-device applications software. Twitter is selected for this study as it is a popular platform for sharing ideas and catching up with news and trends around the world (Overbey et al., 2017). We developed and deployed data extraction software to automatically extract tweet data from Twitter. The program was developed based on Twitter Application Programming Interface (API), which allows users to search for tweets based on specific keywords and geographical location. Full documentation about Twitter API can be retrieved from https://developer.twitter.com/en/docs.

This study focused on analysing public opinions about home schooling in Australia; therefore, we provided a search query (homeschooling OR ‘home schooling’) to the Twitter Search function of the API. In addition, an extra set of parameters, (–45.2310, –9.9191, 112.6062, 153.9182) for minimum latitude, maximum latitude, minimum longitude and maximum longitude is provided to specify a bounding box to focus the search within the geographical area of Australia. There is a quota limit for access to Twitter API, which only allows for a proportion of all available tweets in the latest 7 days to be retrieved. Although not all available tweets are included, the collected tweets are randomly sampled from all available ones, and thus reliable to capture common patterns and trends among public. We ran the data collection program three times, for the weeks commencing 13, 20 and 27 April 2020 to collect the tweets posted during this 3-week period. In total, 10,421 tweets relevant to homeschooling posted in Australia were collected. The number of tweets for weeks 1, 2 and 3 are 2197, 3248, and 4976, respectively. We adopt both quantitative (descriptive) and qualitative approaches to analysing the contents of the collected tweets to identify their major themes and concerns of the Australian public in relation to home schooling during the pandemic.

## Findings

### Phase 1

The first author read all 10,000+ tweets twice before developing the broad codes detailed in column 1 of Table 2. After the second author agreed with the codes, a coding protocol was followed whereby the first author (‘coder 1’) and an independent and qualified non-author (‘coder 2’) independently coded all tweets in the three CSV files by assigning one number/code to each tweet. Inter-coder reliability was computed using Holsti’s (1969) method – based on the percentage of agreement between the two coders. Inter-coder reliability scores were all above 80% (86%, 84% and 89% for weeks 1–3, respectively), thereby confirming that both coders were interpreting the material in a consistent manner. Discrepancies were discussed and codes were revised, yielding a final coding result.

**Table 2.** Australian tweet themes and frequencies (April 2020).

Tweet content (coded)	Week 1 (%)	Week 3 (%)	*z*-score	*p*-value*
1 General and/or neutral tweets	13	12	1.205	0.226
2 Positive tweets	16	11	5.896	0.000
3 Negative tweets	7	12	–6.365	0.000
4 Humorous tweets	26	15	11.122	0.000
5 Tips/advice/sharing of resources	12	10	2.508	0.012
6 Appreciation for teachers	3	7	–6.682	0.000
7 Tweets aimed at Government(s)/politician(s)	10	6	6.001	0.000
8 Social impacts of remote learning	5	8	–4.557	0.000
9 Definition (not home schooling!)	8	19	–11.811	0.000

* significant at *p*-value ⩽ 0.01.

What Table 2 suggests is that over the course of 3 weeks, leading up to and including the commencement of term 2, the ‘novelty factor’ wore off surprisingly quickly (5% reduction in positive tweets) and the public began to lose its sense of humour (humorous tweets dropped 9% off a high base and negative tweets almost doubled to 12%). Appreciation for teachers more than doubled – off a low base (to 7%), while tweets aimed at the Government/politicians halved (to 6%). Surprisingly, the most dominant theme in week 3 is frustration at calling remote learning ‘homeschooling’, which has skyrocketed (from 8% to 19%), further confirming that the novelty has indeed worn off and that the public is becoming increasingly frustrated. *Z*-tests were performed and verified that except for general and/or neutral tweets and tips/advice/sharing of resources, the proportional differences between the two periods of other items are statistically significant at *p*-value ⩽ 0.01.

### Phase 2

The second phase of data analysis was interpretive in nature. The first author went through the coded CSV files again and shaded the more illuminating, illustrative and unique tweets in six of the nine themes. These were then extracted and placed into a MS Word document for further analysis.

#### Positive

Many people commented on the positive effects of being in isolation and homeschooling. They appreciated the perception of ‘slowing down’, being less busy with fewer distractions:

Extended family time. Normally my bad habit is being too busy for too much family time. Now I’m largely responsible for homeschooling the kids while my wife works days so I’m learning and enjoying the time. The kids are too.

Many parents commented on how homeschooling has presented them with the time and opportunity to get to know their children better and appreciate them as individuals – ‘Find the silver lining. With difficulty there is blessing. I can be with my girls 24-7. Precious’, and another, ‘I’m reminded the days are long the years are short’. The exposure to a new learning approach has gained positive reactions. Many respondents commented on the positive results of homeschooling. ‘I know one kid who has a problematical classroom record but is blossoming under home schooling to the extent that his parents are exploring options after the #lockdown ends. And they’re not home-schooling nutters’. Many parents and children are engaged in their learning, having fun and even thriving. ‘Home schooling positive: Teenage son whose reports invariably add “too easily distracted” is getting his work done’. This could be as a result of the homeschooling model being an antithesis of all the negative factors of face-to-face schooling. ‘Winning! Plus, no bags/lunches to pack or school drop off/pick up! #Homeschooling suits me!’ Many parents used the Twitter platform to reach out to others. There is a need to connect as adults experiencing a new challenge and to share experiences and humour. ‘Parents are homeschooling – especially I suspect in lower grades. We don’t need to get bent out of shape about it it’s just a necessity right now’. ‘. . . Getting a bit nostalgic about our office door . . . Offices-remember those?’ ‘SEND COFFEE PLEASE . . .’. Twitter allowed parents to share resources. A collective empathy among parents is evident. The sharing of tips, links, websites and so on is a common thread through the Twitter feeds. ‘Some great tips if you’re a parent home schooling your kids’ https:xxx’. ‘Great ideas and books for parents to help children along with reading writing #wfh#homeschooling https:xxx’.

#### Humour

Twitter is also a platform for sharing humour about homeschooling. Parents embraced humour as a coping mechanism. ‘. . . not sure I’m managing the work/life/homeschooling balance quite right. There’s going to be a very cranky “teacher” in our house tomorrow morning!!’; ‘It turns out I really suck at Grade 5 math’s. #homeschooling’. It acts as an adult forum for venting frustration and a feeling of being overwhelmed. ‘1 hour into day 2 of home schooling and I’ve already decided it’s time for a fire drill. #Homeschooling2020#getthemout’. Humour on Twitter is allowing parents to express themselves safely without fear of recrimination. They have an audience who are experiencing similar challenges. It is also a platform for people in isolation as it is instant communication without having to leave home. Many Twitter comments mentioned alcohol consumption. ‘Well that went well. Bwahahaha . . . sob . . . I need a drink. #homeschooling #workfromhome #completelybloodyincompatible’.

#### Negatives

Many negative aspects of homeschooling were highlighted in the tweets. The platform was used by many to express their anxiety and frustration. ‘. . . I talk to frustrated parents every day. I’m bloody frustrated and exhausted and angry too’. There is a strong suggestion of not coping and relationship deterioration. ‘I honestly could not do homeschooling for a term. My son would suffer academically and our relationship would suffer’. The situation has also highlighted the divide between public and private schools. ‘If kids are behind in literacy & numeracy it’s because this govt fails to invest enough in our public schools’. ‘. . . Covid 19 coronavirus RICH PEOPLE PROBLEMS’. ‘Why is every home schooling case study on ABC in a relatively well-off household? What about the poor kids @abcnews?’ Twitter users express a frustration with technology/lack thereof. ‘No internet connection this morning . . . home schooling is canceled for today’. ‘So, we had electricity issues on Thursday in my area so we couldn’t log in and partake in the online learning . . . I’m so deflated’. The platform was also used to express concerns regarding gender inequality. ‘Mostly it falls to the women in the household making it more difficult for keeping their job let alone career progression’. ‘The #GenderPayGap will have a shocking impact on our critical #COVID19 workforce ‘who will do the lion’s share of home schooling and child caring during #iso when both parents are normally at work whose job will get priority? His/He earns more’. ‘Apparently mums struggling with juggling looking after a house working being a mum 24/7 and home schooling during isolation means they hate their kids’. Twitter users also picked up on how ‘homeschooling’ has exposed the vulnerable in society. ‘Some children are exposed to crap parental behavior including actual abuse some don’t have multiple bedrooms/laptops/ backyards to make the home schooling thing a pleasant and productive experience. I feel for kids the most’. ‘Dear parents of autistic/adhd kids who are homeschooling during lockdown. How are you coping? How are your kids coping with . . .?’ ‘If kids had online home schooling there would be no bullying in schools which is an epidemic’. Mental health challenges are also exposed: ‘I’m not helping with my daughter’s home schooling. It’s very hard. Depression doesn’t help in the mix either’. Twitter also points to challenges in Australian society as a whole, ‘People have lost homes in the bushfires. People have lost jobs due to the lockdown. Parents struggle with homeschooling . . .’.

The negative influence of technology is argued on Twitter. ‘It’s like a child being raised by robots’. ‘This home schooling thing is a great way to get kids addicted to screens isn’t it’. ‘. . . It’s not the work it’s the endless portals passwords and IT administrative confusion’. For parents, the reliance on technology tests their parenting values and approach:

Home schooling is a #BigTech wet dream. We have a 9 year old now with a google account something we would not have waved through until he was 16 but to deny means he has no interaction with his education and school mates.

Twitter is also a platform for raising concerns about the demands made on parents’ time and balancing the demands of their children’s learning and their own work commitments. ‘Deliberating what the appropriate time to start making work calls in this COVID-19 time when the person I’m calling is isolating and home schooling?’ ‘I’m getting family help with my kids as home schooling with this job is a struggle’. ‘I’m not saying u can’t have 2 parents work but children’s needs have 2 come 1st’.

#### Appreciation for teachers

Many Twitter users expressed their appreciation and respect for teachers, particularly by week 3, suggesting that the role of teaching has till now been undervalued. ‘I am so sorry, I and many other parents have seen 1st hand how tough the job is for teachers’. ‘When COVID-19 restrictions are lifted I demand ALL good teachers get paid DOUBLE . . .’. ‘It’s fair to say that since this crisis began most parents have discovered a newfound respect for teachers’. Having to adopt the role on a ‘temporary’ basis has revealed the demands on teachers. Many did not realize the effort involved in teaching. ‘. . . Teaching is a demanding occupation homeschooling might be revealing to many parents just how difficult it is’. ‘. . . homeschooling has taught parents anything it’s that it takes a specific kind of person to have the patience and dedication to teach’. Some Twitter users refer to teachers as ‘heroes’. ‘Teachers are unsung heroes until everyone has tried home-schooling. We owe them thanks along with delivery workers’. For many, teachers play important part in their children’s lives. Twitter allows users to express their emotions about the relationship, ‘. . . I got a bit teary. They are so supportive and realistic and I love them and now I’m sad all over again that my boys are out of their care for this term’.

#### Government and politicians

While Twitter users express respect and affection for teachers, the vitriol directed at the country’s leaders is largely negative. Many Twitter comments concerned the government’s response and communication regarding education and the pandemic. It is a platform for venting frustration and anger towards politicians, at times through sarcasm. The primary concern is the miscommunication and confusion around the messages presented to parents by the governments. ‘Welcome to COVID-19 Oz style . . . so we have the Federal government telling us that we should be sending kids to school yet state govts are saying only send them if absolutely necessary . . . would you please clarify’. ‘What a fuckstick. It’s like getting a lecture from a drunk uncle. Incoherent ramblings and repeating himself. Speech writers from the IPA?’ The issue is also around poor leadership. ‘@Dan TehanWannon showed some pretty poor leadership with his outburst. Expect more from elected officials. Political squabbling is pathetic at this time’.

‘PM urges teachers not to force parents into choice between home schooling and food on the table’ suggests fear of the unknown. Parents are concerned about the health risk to their children of returning to school and hold politicians responsible, ‘if you force schools to open I will unenroll my child from school and register them for home schooling . . .’.

#### Definitions

The definition of homeschooling/remote learning elicited many emotional responses. Many tweets correctly noted that it is not homeschooling, rather learning from home or remote learning. ‘Every time people refer to it as home schooling I want to scream. It’s not bloody homeschooling you morons’ and ‘parents are merely fulfilling a temporary, supervisory role, and are not expected to teach’. Comments acknowledged that teachers are the professionals who are still teaching. ‘Hey again for the people in the back it’s NOT #homeschooling it’s learning from home using stuff professional teachers have prepared’. Another user summarized the role:

Australian parents are supporting students as they learn at home. Teachers have designed the curriculum, planned lessons, decided on assessment and will mark student work. Parents are definitely working hard to support learning, but they are not homeschooling their children.

The language around learning is confusing and varied: remote learning, distance learning, supporting students learning at home, online learning. ‘It’s not outrage at the wrong term it’s frustration and misrepresentation. Distance education is the right term. Home schooling it is not’. Arguably, the frustration over the definition of the learning is highlighting how unsure parents feel in this new role:

I came across some online twitter debate on whether it was technically home schooling when in reality you aren’t setting the work. After 3 hours of helping my 11 year old navigate math’s questions I will call it whatever the fuck I like . . . exhausting mostly lol.

Another user stated, ‘We know it’s not home schooling. If it’s easier for parents to call it that and it makes them feel better then so be it’. Finally, a humorous take on the definition:

You can call it Home Schooling if it comes from the Homeschoole region of France otherwise it is just sparkling domestic education.

## Conclusion and implications

COVID-19 is the biggest health, social and economic emergency the world has faced since the Second World War – and its consequences will endure for years to come (Khan, 2020). In response, Australia’s national cabinet process has worked effectively by building confidence and trust between jurisdictions and cutting through narrow partisan politics in the name of ‘public interest’. Different levels of government have had to come together to negotiate, largely as equals, resulting in better policy than if one level had simply dictated terms (Smith, 2020). For example, the actions of the NSW and Victorian governments on the second weekend in March, where they pushed a Stage 3 lockdown ahead of a reluctant commonwealth have been shown to be correct. Likewise, the actions of Queensland in imposing quarantines on domestic travellers were followed by the NT, WA, SA and Tasmania and have effectively stopped the spread of the virus between states. Interestingly, the *only* major split in the initial COVID-19 response has been over schools, an area that is funded dually and where the commonwealth has tried to use funding to exert control over the states’ responsibilities. This school split has frustrated and confused an already anxious public, who, quite justifiably turned to social media to voice their frustrations. A single recommendation emanating from this study is that the Australian federal and state governments should agree on a school attendance/remote learning policy *before* the next pandemic or other crisis strikes. In other words, have a national/state policy ‘on the shelf’.

It is also highly likely that hybrid approach to primary and secondary education will emerge post-COVID. In other words, parts of the curricula could transition online over time. This, coupled with the very high likelihood of future pandemics and crises, will no doubt need to be reflected in teacher recruitment and training going forward and in school policies and practices pertaining to education during crises.

It will be interesting to see how many of the aforementioned issues play out in real time in the United States this fall. Schools there are about to return from their long summer break and tensions are escalating between education secretary Betsy DeVos and numerous state governors. Early indications are that the US model for the fall term and 20/21 academic year is likely to be partisan, heterogenous, probably hybrid and possibly quite politically divisive.

Like all similar studies, this one is not without limitations. The analysis was carried out on a relatively small data set (10,000 tweets) and for a short time period (3 weeks). The data were also collected from a single social media platform (Twitter). Nevertheless, the findings shed light on the emerging issues and challenges in the context of remote learning which are confronting policy makers, politicians and principals alike. In addition to the textual content, other meta-data, such as profile of Twitter user (e.g. location, description, number of followers, number of following), hash-tag, mentioned users, number of likes, number of re-tweets and links to other websites are also available. However, as a preliminary study, our analysis was mainly focusing on the textual content of the tweets to study the topics being discussed by the Australian public at a point in time.

Due to the unrestricted boundary of social media, the analysis can be extended to other countries, such as New Zealand and beyond, for comprehensive insights. While this study focused on Twitter, analyses of Facebook and Instagram would be most worthwhile too. Future studies may also consider employing quantitative approaches with text mining, sentiment analysis and topic modelling (Silge and Robinson, 2017) to effectively process and analyse large-scale social media data sets. Future research might employ a choice modelling technique, such as best-worst scaling, to determine what learning approaches parents deem most and least appealing, for it is also possible that traditional (i.e. voluntary, non-forced) home schooling may increase in popularity following some parents/pupils’ positive experiences during the pandemic.
